# Extracellular Vesicles: A Platform for the Structure Determination of Membrane Proteins by Cryo-EM

**DOI:** 10.1016/j.str.2014.09.005

**Published:** 2014-11-04

**Authors:** Tzviya Zeev-Ben-Mordehai, Daven Vasishtan, C. Alistair Siebert, Cathy Whittle, Kay Grünewald

**Affiliations:** 1 Division of Structural Biology, Wellcome Trust Centre for Human Genetics, University of Oxford, Oxford OX3 7BN, UK

## Abstract

Membrane protein-enriched extracellular vesicles (MPEEVs) provide a platform for studying intact membrane proteins natively anchored with the correct topology in genuine biological membranes. This approach circumvents the need to conduct tedious detergent screens for solubilization, purification, and reconstitution required in classical membrane protein studies. We have applied this method to three integral type I membrane proteins, namely the *Caenorhabditis elegans* cell-cell fusion proteins AFF-1 and EFF-1 and the glycoprotein B (gB) from Herpes simplex virus type 1 (HSV1). Electron cryotomography followed by subvolume averaging allowed the 3D reconstruction of EFF-1 and HSV1 gB in the membrane as well as an analysis of the spatial distribution and interprotein interactions on the membrane. MPEEVs have many applications beyond structural/functional investigations, such as facilitating the raising of antibodies, for protein-protein interaction assays or for diagnostics use, as biomarkers, and possibly therapeutics.

## Highlights

• Intact membrane proteins with correct topology in genuine biological membranes
• Display system circumvents need for detergent solubilization and reconstitution
• 3D reconstruction of proteins in the membrane by subvolume averaging
• Mapping spatial distribution and relationships between proteins on the membrane

## Graphical Abstract

**Figure:**
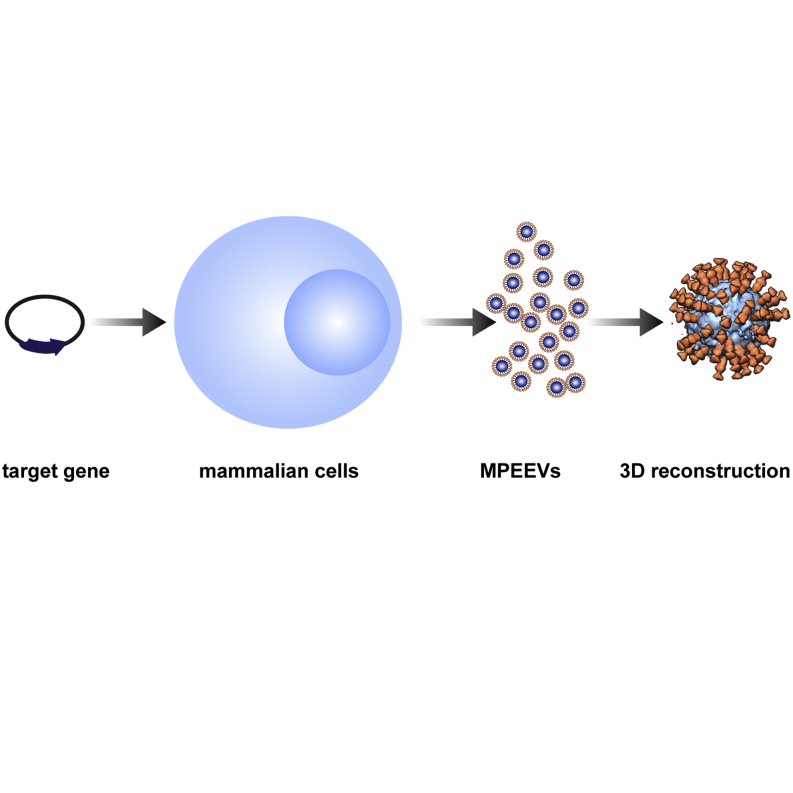

## Teaser

An elegant approach to display target membrane proteins on cell-derived vesicles in the topologically correct orientation is presented. Structural analysis by cryo-EM of those vesicles allowed reconstruction of the target proteins and mapping the spatial distribution on the membrane.

## Introduction

Membrane proteins are a central subclass of the proteome (Wallin and von Heijne, 1998). They are involved in many essential biological processes, including cell signaling, cell adhesion, transport across the lipid bilayer, transduction of energy, and immune response. As such, membrane proteins are implicated in many disorders and are key targets for diagnostics and therapeutics. Prerequisite to conducting any research into membrane protein function is the successful production of the protein of interest in a functional form. Producing intact membrane proteins is an inherently challenging task due to their requirement for a lipid environment, and while remarkable achievements have been made in the past several years toward the production of membrane proteins, the requirement for lipidic environment remains a severe restriction to the structure determination of these otherwise desirable targets (Moraes et al., 2014). Most procedures developed involve isolating the protein by detergent solubilization, followed by a purification step and subsequent reconstitution into an artificial membrane e.g., liposomes, bicelles, or nanodiscs (Denisov et al., 2004, Whiles et al., 2002). These procedures are highly time consuming and suffer from further drawbacks, including low yields and high cost. Perhaps most importantly, preserving the correct topology of membrane proteins is often crucial for their function but is very difficult to achieve during reconstitution experiments. Additionally, the biological relevance of in vitro model systems is limited by the relative simplicity of the lipid composition of the artificial membranes when compared to native membranes that comprise a considerably more diverse range of lipids, often with specific ratios that can also form local subdomains (Simons and Ikonen, 1997).

Membrane enveloped viruses have been successfully used as a platform for displaying intact membrane proteins on their surface. This approach is referred to as pseudotyping, a process in which the native virus surface protein is replaced with the protein of interest. This gives rise to membrane proteins that are properly folded and oriented on cell-derived membranes. Vesicular stomatitis virus (VSV) is a favorable platform for the pseudotyping approach with well-demonstrated success (Whitt, 2010). Simpler systems that circumvent the related biosafety laboratory requirements for work with VSV pseudotypes are virus-like particles (VLPs) that have been likewise applied successfully to display membrane proteins (Noad and Roy, 2003). However, an inherent limitation of the virus-based and VLP approaches is the need for viral components. Additionally, integral membrane proteins with bulky cytoplasmic domains will not be readily packed into either pseudotyped viruses or VLPs due to steric hindrances from the virus capsid or matrix proteins. Furthermore, in these cases the cytoplasmic domain of the membrane protein is potentially altered.

With the aforementioned limitations in mind, we have developed an alternative approach that provides high yields of cell-derived, membrane protein-enriched extracellular vesicles (MPEEVs). The basis for this approach is the utilization of the recently characterized biological process of membrane vesicle secretion (György et al., 2011). Extracellular vesicle secretion seems to be a universal and evolutionary conserved process under both physiological and pathological conditions. Chemical vesiculants like paraformaldehyde in combination with dithiothreitol can induce release of giant plasma membrane vesicles. However, these agents have severe effects on the integrity of the proteins and thus often limit the use of such preparations to study membrane biophysics (Sezgin et al., 2012). The here presented approach does not require any vesiculants or viral components.

## Results and Discussion

### MPEEV Production and Characterization

To produce extracellular vesicles enriched with a specific membrane protein, adherent mammalian cells were transfected with the gene corresponding to the full-length protein of interest in a standard expression vector using an actin promoter. The overexpression of the protein resulted in the accumulation of MPEEVs in the growth medium (for details, see the Experimental Procedures). The source compartment of the vesicles might vary from protein to protein. The MPEEVs were then separated from producer cells by differential centrifugation of the supernatant.

**Figure 1:**
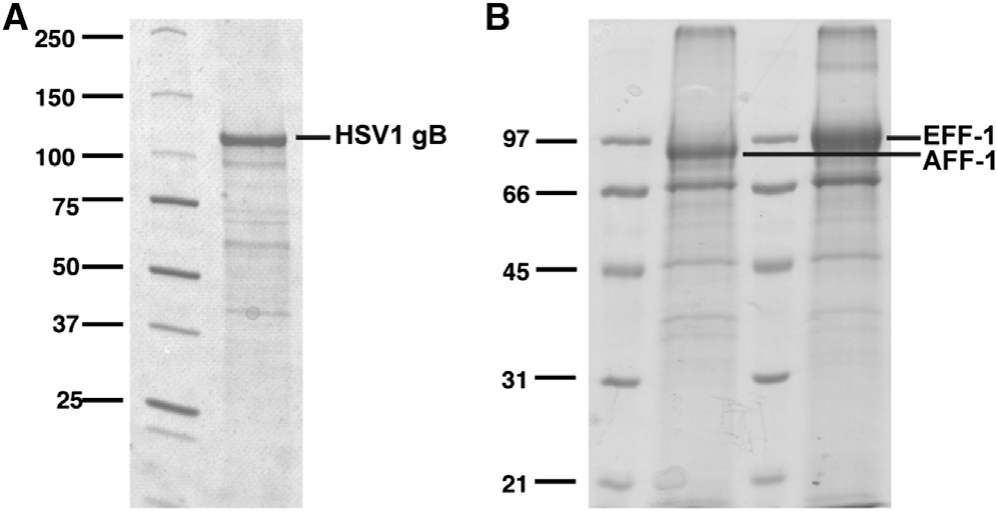
Analysis of MPEEVs by SDS-PAGE (A) Vesicle preparation with Herpes simplex virus 1 (HSV1) glycoprotein B (gB). gB appears as prominent band at a molecular weight of ∼110 kDa. (B) Vesicle preparation with the *C. elegans* fusion proteins EFF-1 and AFF-1, the proteins appear as predominant bands at a molecular weight of ∼97 kDa.

We have applied this method to three integral membrane proteins, namely the *Caenorhabditis elegans* cell-cell fusion proteins AFF-1 and EFF-1 and the glycoprotein B (gB) from Herpes simplex virus type 1 (HSV1). To estimate the relative enrichment of the corresponding membrane protein in the MPEEVs, an aliquot of the vesicle preparation was loaded on SDS-PAGE (Figure 1). While some contaminants were observed, a major band at the expected molecular weight was clearly apparent for each of the three different samples. Based on the SDS-PAGE, the estimated yield from one T175 flask was 50–100 μg of protein. To further quantify the relative enrichment and analyze the nature of the contaminants, the MPEEVs were analyzed by mass spectrometry using an exponentially modified protein abundance index (emPAI) (Table 1). The emPAI analysis verified that the corresponding membrane protein of interest is the most abundant protein in the respective vesicle preparation, correlating well with the SDS-PAGE results. Importantly, no other membrane proteins were detected and most of the other proteins found were contaminants, either from the serum added to the cell culture medium or from the transfection reagent.

**Table 1:** Major Proteins Identified by Mass Spectrometry in the Vesicles Preparations and Their Relative Abundance

Protein Name^a^	NCBI Accession	Mass (Da)	HSV1 gB			AFF-1			EFF-1		
			Score^b^	emPAI^c^	Rel. Abund.^d^ (%)	Score^b^	emPAI^c^	Rel. Abund.^d^ (%)	Score^b^	emPAI^c^	Rel. Abund.^d^ (%)
Envelope glycoprotein B (HSV1)	1353200	100,875	16,873	70.91	27.6						
Protein AFF-1 anchor cell fusion failure-1 (*C. elegans*)	193204255	68,617				974	2.07	33			
Protein EFF-1, isoform a (*C. elegans*)	71982882	75,494							1004	1.66	16
Histone H2B homolog	156371481	24,545				225	1.78	28	392	1.78	17
Actin family^e^	178045	26,147	2,918	46.51	18.1	221	1.05	17	185	0.35	3
Hemoglobin fetal subunit beta	62460494	15,963	998	33.03	12.9	95	0.78	12	234	1.62	15
Pyruvate kinase PKM	146345448	58,378	2,081	18.19	7.0						
Tubulin beta-3 chain	12963615	50,842	1,422	17.00	6.6				134	0.37	3
14-3-3 protein zeta	82197807	27,929	625	16.42	6.4						
Ras-related protein Rap	75077355	21,040	356	13.03	5.1						
Annexin A2	113951	38,937	1,730	12.89	5.0						
T-complex protein 1 subunit zeta	115305833	58,376	405	10.15	4.0						
Class-III intermediate filaments	138535	53,754	1,500	9.64	3.8						
Tubulin alpha	116256086	50,804	1,614	9.10	3.5						
Histone cluster 1, H2ag-like	291410763	27,347							141	1.00	10
Serum albumin	1351907	71,244				152	0.20	3	282	0.31	3
Galectin-3-binding protein	81861611	65,270				272	0.41	7	315	0.63	6
60S acidic ribosomal protein P2	133062	4,692							72	0.79	8
40S ribosomal protein SA-like	296190805	32,906							182	0.47	4
Guanine nucleotide-binding protein subunit beta-2-like 1	5174447	35,511							170	0.56	5
Glyceraldehyde-3-phosphate dehydrogenase-like	488563203	35,942							190	0.55	5
Laminin-binding protein	34234	31,888							197	0.49	5

a All proteins other than gB of HSV1 and AFF-1 and EFF-1 of *C. elegans* are originating either from the BHK cells used to produce the vesicles or contaminations, either from the serum added to the cell culture or transfection reagent.

b Mascot score for confidence of protein identification is defined as the –log value of the probability P that this assignment is made by chance (Mackeen et al., 2010).

c Exponentially modified protein abundance index (emPAI) analysis (Ishihama et al., 2005, Trudgian et al., 2011).

d Relative abundance (Rel. Abund.) in respect to the proteins with the highest emPAI listed in this table.

e Actin family representing gamma-actin, cytoplasmic actin 2, actin-cytoplasmic 1-like.

**Figure 2:**
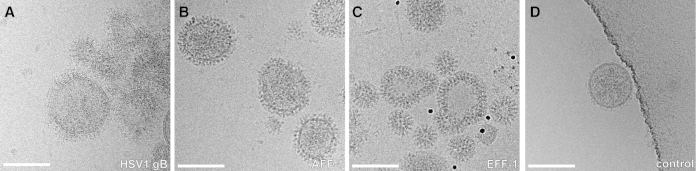
Visualization by of MPEEVs by Cryo-EM Projection images of vesicles collected from the culture medium of cells transfected with the expression plasmid for full-length HSV1 gB (A; defocus −3 μm); full-length AFF-1 (B; defocus −5 μm); full-length EFF-1 (C; defocus −5 μm) and cytosolic YFP (D; defocus −5 μm). Scale bar represents 100 nm.

To characterize the protein incorporation in the membrane, the vesicles were imaged with electron cryomicroscopy (cryo-EM) and electron cryotomography (cryo-ET). The size and morphology of the vesicles were dependent on the displayed protein (Figure 2). For HSV1 gB and AFF-1, the vesicles were mostly spherical and ∼100 nm in diameter. EFF-1 vesicles were generally smaller in diameter and had variable morphologies. In the case of HSV1 gB, we observed elongated spikes protruding from the membrane (∼16 nm; Figure 2A). In the case of AFF-1 and EFF-1, the vesicle membranes were uniformly covered with an ∼12–14 nm thick protein layer that appeared to consist of discrete densities protruding radially outward from the membrane (Figures 2B and 2C). In control vesicle preparations, where the cells were transfected with an expression plasmid for cytosolic yellow fluorescence protein (YFP), notably ∼100× less vesicles were secreted. These vesicles could be seen to display only very small, extra-membranous densities, which were clearly different from those observed for the MPEEVs (Figure 2D versus Figures 2A–2C). This indicates that our experimental system is highly suitable for specifically displaying topologically correct membrane proteins, and vesicle secretion is induced by the expression of the membrane proteins. Furthermore, to demonstrate the stability of these preparations, we have successfully imaged vesicles after storage in buffer at 4°C for over 2 months without noticing any decay. This characteristic might be crucial for a number of nonstructural applications.

### Structure Determination from MPEEVs

**Figure 3:**
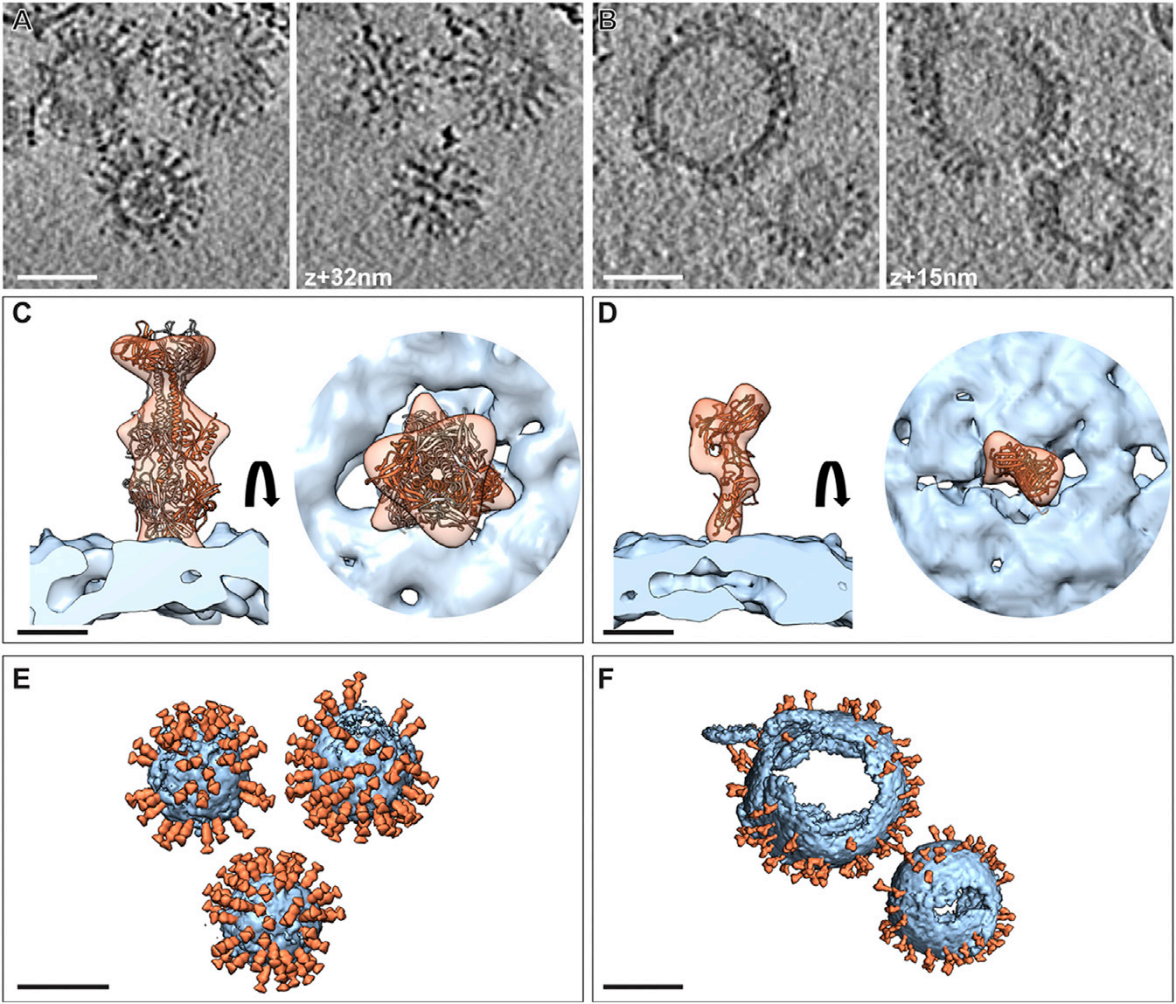
3D EM Reconstruction of the Proteins on the Membrane (A and B) Central and tangential slices through a tomogram of MPEEVs displaying gB (A) and EFF-1 (B). Scale bar represents 50 nm. (C and D) Isosurface representation of the sub-volume reconstruction of gB with the trimer crystal structure (Protein Data Bank [PDB]: 2GUM) fitted (C) and EFF-1 EM map with a protomer of EFF-1 crystal structure (PDB: 4OJC) flexibly fitted (D), side (left) and top views (right) are presented. Membrane is shown in light blue; protein in orange. Scale bar represents 5 nm. (E and F) Isosurface representations of the tomograms shown in (A and B). The subvolume reconstruction and the membrane were placed back into the determined position and orientation of individual protein spikes such enabling analysis of relative orientations and interactions. Scale bar represents 50 nm. See also Movies S1 and S2.

To generate a 3D reconstruction of the membrane proteins in the context of the membrane we have applied cryo-ET with subsequent averaging of tomographic subvolumes. Subvolume averaging is a method of aligning and averaging a large number of extracted volumes that contain the structure of interest, in order to greatly improve the signal-to-noise ratio (Briggs, 2013, Förster et al., 2005). For the EFF-1 and gB structures (Figure 3), several hundred subvolumes were automatically picked at the vesicle surfaces using a local minimum search. These volumes were then iteratively aligned and averaged in an unbiased, reference-free manner. The resulting 3D reconstruction of the elongated spikes observed for gB showed a 3-fold symmetry and was very similar to the postfusion crystal structure of gB (Heldwein et al., 2006) and the 3D reconstruction of the ectodomain bound to liposomes (Maurer et al., 2013) (Figure 3C). The resulting 3D reconstruction of natively-anchored EFF-1 had an asymmetric elongated shape (Figure 3D). For further details on the EFF-1 3D reconstruction, see Zeev-Ben-Mordehai et al. (2014). In that study, it was shown that vesicles could have substantially different morphologies, depending on the protein displayed and the time point of harvest. Furthermore, imaging of vesicles undergoing fusion revealed highly elusive membrane rearrangements occurring during AFF-1- and EFF-1-mediated fusion (Zeev-Ben-Mordehai et al., 2014).

Subvolume averaging, in addition to the 3D structure, provides the means to analyze the membrane proteins spatial distribution and to probe whether higher order assemblies are apparent. This analysis was performed by subsequently back-plotting the average structure into orientations and positions at which the proteins were found on the membrane thus enabling to visualize and assess the relative protein topology and interprotein interactions (Figures 3E and 3F; Movies S1 and S2 available online). For the examples presented, no ordered lattice or preferred intermolecular interaction was observed in the case of EFF-1. In the case of HSV1 gB some patches of trimers showed preferred lateral interaction presumably mediated by the trimer midregions as observed earlier for the soluble gB ectodomain upon interaction with artificial liposomes (Maurer et al., 2013).

In summary, the experimental system described here works independent of detergents, vesiculants, and viruses, and as such, we envisage MPEEVs being used in a broad number of applications. As the vesicles originate from cells, the intact membrane protein is embedded in membranes with a native lipid composition. MPEEVs can be potentially isolated from a wide variety of cell-wall free cell types. The success in MPEEV production relies on the overexpression of the protein of interest and as a high level of expression is crucial, plasmids using a strong promoter are required. The optimum time, posttransfection, for collecting the MPEEVs will vary for different proteins and needs to be determined case by case. However, this optimization is very fast and high quality material can be produced within a week as opposed to several months or even years required for solubilization and reconstitution-based approaches. Here, results are presented for three type I transmembrane proteins, the extent to which this method is applicable for studying other families of membrane proteins remains to be determined. As demonstrated here, the coupling of this experimental system with techniques that are ideally suited to study proteins within biological membranes such as cryo-EM and cryo-ET enabled structural characterization of the membrane protein of interest. Additionally, MPEEVs can be used as highly protein-enriched, semipurified starting material for classical detergent-based purification approaches currently applied for structure determination by crystallography or NMR. The applications for MPEEVs, however, are not limited to structural determination. They can credibly be used for a wide range of exciting applications including antibody generation, protein-protein interaction assays, diagnostics use, biomarkers, and possibly therapeutics.

## Experimental Procedures

### Vesicle Preparation

Adherent Baby Hamster Kidney cells (BHK-21, clone 13, ECACC 85011433) were grown in a T175 flask; at ∼70% confluency, they were transfected with either *aff-1* (Avinoam et al., 2011), *eff-1A* (Avinoam et al., 2011), or HSV1 gB (Pertel et al., 2001) gene in pCAGGS plasmid using Lipofectamine (Invitrogen). Following a 2 hr incubation at 37°C and 5% CO_2_, the medium with the transfection reagent was removed and replaced by 2% FBS/GMEM-CM (Invitrogen). Transfected cells were allowed to grow for 24 hr or 48 hr at 37°C and 5% CO_2_. Following the incubation, the medium was collected and cleared from cell debris by centrifugation at 3,000 × *g* for 20 min and 4°C. The vesicles were pelleted through a 20% sucrose cushion at 100,000 × *g*, and resuspended in 25 mM HEPES pH 7.4, 130 mM NaCl. For AFF-1 and EFF-1 vesicle preparations each 19 independent repeats were performed for gB 11 independent repeats were performed. For every vesicle preparation, 2 aliquots were analyzed by cryo-EM.

### Mass Spectrometry Analysis

For the analysis of protein composition, vesicles were precipitated using chloroform/methanol as described previously (Wessel and Flügge, 1984) followed by in-solution trypsin digestion (Xu et al., 2008). The mass spectrometry analysis was performed by nano ultraperformance liquid chromatography tandem mass spectrometry (UPLC-MS/MS) using a nano Acquity UPLC system coupled to a QTOF premier (Waters) as described previously (Mackeen et al., 2010). MS/MS spectra were searched against the National Center for Biotechnology Information (NCBI) database and proteins identified quantified in a relative fashion using the empirically modified abundance index (emPAI) approach as described (Ishihama et al., 2005, Trudgian et al., 2011) (Table 1). For each vesicle type (gB, EFF-1, AFF-1), mass spectrometry analysis was performed from two independent preparations and found to be highly similar. The data given in Table 1 is the result from one of these experiments.

### Electron Cryo Microscopy Data Collection

Microscopy was performed at either 200 keV or 300 keV using a TF30 “Polara” electron microscope (FEI) equipped with a QUANTUM 964 postcolumn energy filter (Gatan) operated in zero-loss imaging mode. A 50 μm C2 aperture and a 20 eV energy-selecting slit were used. Projection images and tilt series were recorded on a 4 k × 4 k CCD camera or K2 summit direct detector at a nominal magnification of 95,000× or 77,000× resulting in a calibrated pixel size of 0.38 nm or 0.28 nm at the specimen. Tilt series were collected at 200 kV or 300 kV using SerialEM (Mastronarde, 2005) at a defocus of –2 μm in 3° or 4° increments covering an angular range from –60° to 60°. The total electron dose for the tilt series was kept between 60 and 80 electrons/Å^2^.

### Tomographic Reconstructions

Tomographic reconstructions were calculated in IMOD (Kremer et al., 1996) using weighted back-projection (Sandberg et al., 2003).

### Subvolume Picking

Subvolumes were picked using a local minima search on 4× binned and Gaussian-filtered versions of the tomograms as described (Zeev-Ben-Mordehai et al., 2014). In brief, all local minima with intensities lower than 2 SD below the mean, and within ∼150 Å of the manually segmented vesicle membrane, were considered as particles to be averaged, resulting in 1,973 subvolumes for EFF-1 and 1,380 subvolumes for gB. Initial orientations of the sub-volume “boxes” were approximated as normal to the membrane. Using PEET version 1.9 (Nicastro et al., 2006), five iterations of alignment against the initial average of all 1,973 subvolumes were performed on unmasked particles, in order to refine the picking while aligning the membrane as well as the particle. For EFF-1, the 801 subvolumes giving cross-correlation scores above the mean were subsequently used for subvolume averaging. For gB, 748 subvolumes from the two tomograms giving the most consistent averages were used. Using UCSF Chimera (Pettersen et al., 2004), the orientations and positions of these subvolumes were visualized concurrently with the filtered tomogram maps to validate the results of the picking process.

### Subvolume Averaging

Averaging was performed using PEET version 1.9 (Nicastro et al., 2006). The picked subvolumes for each protein were split into two evenly sized groups (based on even and odd particle indices) for the averaging and the final FSC calculation. For each of these four groups (two for EFF-1, two for gB) the average of all particles in the group was used as the initial template. Six iterations of refinement of the positions and orientations with successively finer sampling increments while including progressively higher spatial frequency information were applied, with the particles masked to remove the membrane and neighboring particles. The resulting structures from the two independent refinements were aligned and the resolution determined using Fourier shell correlation (FSC). The gB reconstruction went through four more iterations of refinement with 3-fold symmetry applied.

The final structure, created by refining and averaging all 801 subvolumes for EFF-1 and all 748 subvolumes for gB, was low-pass filtered using a Gaussian curve with a width matching the FSC curve. The membrane structure from the initial picking and the final particle structure were then plotted back into their relative positions on the original tomogram.

UCSF Chimera (Pettersen et al., 2004) was used for visualization, rigid body fitting of the crystal structure, and preparation of the figures.

## Author Contributions

T.Z. and K.G. designed the experiments. T.Z. and C.W. performed the experiments. T.Z., D.V., C.A.S., and K.G. processed and analyzed the data. T.Z. and K.G. wrote the manuscript, and all authors commented on it.

## Supplemental Information

Movie S1. Slice-by-Slice Visualization of Both the Tomographic Reconstructions and the Back-Plotting for HSV1 gB, Related to Figures 3A and 3E

Movie S2. Slice-by-Slice Visualization of Both the Tomographic Reconstructions and the Back-Plotting for EFF-1, Related to Figures 3B and 3F
